# Is There a Way to Break the Glass Ceiling in the Diagnosis and Treatment of Extraintestinal Manifestations (EIMs) in the Course of Inflammatory Bowel Diseases? A Comprehensive Review of Diagnosis, Pathophysiology, and Treatment of EIM

**DOI:** 10.3390/jcm15187159

**Published:** 2026-09-15

**Authors:** Dominik Ruta, Hubert Żywno, Arkadiusz Bielski, Konrad Lewandowski

**Affiliations:** 1 Department of Gastroenterology and Internal Medicine, National Medical Institute of the Ministry of the Interior and Administration, 02-507 Warsaw, Poland; dominikruta7@gmail.com (D.R.); hubert.zywno@gmail.com (H.Ż.); arkadiusz.bielski@pimmswia.gov.pl (A.B.); 2 Doctoral School, Medical University of Warsaw, Żwirki i Wigury 61, 02-091 Warsaw, Poland; 3 IBD Clinical and Research Center, ISCARE a.s., 190 00 Prague, Czech Republic

**Keywords:** inflammatory bowel diseases, extraintestinal manifestations, pathogenesis, diagnosis, treatment, interdisciplinarity

## Abstract

Inflammatory bowel diseases (IBDs), particularly Crohn’s disease (CD) and ulcerative colitis (UC), are chronic disorders that affect not only the gastrointestinal tract but also other organ systems through extraintestinal manifestations (EIMs). It is estimated that EIM may occur in up to half of all patients, with at least two different EIM present in 20% of cases and preceding the diagnosis of IBD by approximately five months in 26% of cases. The most frequently reported manifestations include dermal lesions, spondyloarthropathy, and hepatobiliary disorders, although ocular involvement is also observed, significantly impairing patients’ daily functioning. Given the profound impact on quality of life (QoL) and the associated diagnostic challenges, this article provides an overview of the epidemiology, pathophysiological mechanisms, and current diagnostic and therapeutic strategies for EIM. Special emphasis is placed on the importance of early recognition and coordinated, multidisciplinary care involving gastroenterology, rheumatology, dermatology, ophthalmology, and other specialties. The roles of genetic, immunological, and environmental factors in the development of these complications are also discussed, along with recent therapeutic advances, including biologics and small molecules. Future directions highlight the need for multicenter studies aimed at improving both prophylactic and therapeutic strategies. This comprehensive narrative review aims to summarize current evidence on the epidemiology, pathophysiology, diagnosis, and treatment of extraintestinal manifestations in IBD, with particular emphasis on their early recognition, multidisciplinary management, and clinical implications. We also discuss emerging therapeutic strategies, screening and referral approaches, and areas requiring further research.

## 1. Literature Search Strategy

This article was designed as a comprehensive narrative review. Relevant literature was identified through searches of PubMed/MEDLINE and the Cochrane Library, supplemented by reference screening of key reviews and international clinical guidelines. Searches focused on combinations of terms related to inflammatory bowel disease (“inflammatory bowel disease”, “Crohn’s disease”, “ulcerative colitis”) and extraintestinal manifestations, including musculoskeletal, dermatological, ocular, hepatobiliary, renal, pulmonary, cardiovascular, neurological, psychological, and thromboembolic manifestations. Additional searches were performed for individual therapeutic agents and emerging treatment strategies. Priority was given to international guidelines, systematic reviews and meta-analyses, randomized controlled trials, and large observational studies; case reports and case series were considered primarily for rare manifestations or emerging therapies where higher-level evidence was unavailable. The literature search covered publications available up to [July 2026], with the final search performed on [20 July 2026]. As this was a narrative rather than a systematic review, no formal study-selection protocol, risk-of-bias assessment, or quantitative evidence synthesis was undertaken.

## 2. Introduction

Inflammatory bowel diseases (IBD), comprising ulcerative colitis (UC) and Crohn’s disease (CD), are chronic systemic disorders that may present not only with gastrointestinal symptoms but also with a wide spectrum of extraintestinal manifestations (EIMs) [1,2]. EIMs affects up to 50% of IBD patients and significantly impairs quality of life (QoL) [3]. The most commonly involved systems include the musculoskeletal system, skin, and eyes, although EIMs may also affect the liver, lungs, or pancreas [4]. In some cases, EIMs may precede intestinal symptoms or occur independently of the underlying disease activity [3].

The pathogenesis of EIMs remains incompletely understood. It is hypothesized that EIMs may result from the extension of the immune response from inflamed intestinal tissue to extraintestinal organs, abnormalities in leukocyte trafficking, alterations in the gut microbiome, and genetic predisposition [5]. The management of EIMs poses a clinical challenge, as not all manifestations respond to therapies targeting intestinal inflammation. While certain forms of EIMs, such as non-axial spondyloarthropathy (non-axSpA) or erythema nodosum (EN), may improve with control of gut inflammation, others—including primary sclerosing cholangitis (PSC) and ankylosing spondylitis (AS)—often require independent therapeutic strategies [6].

Effective management of EIMs is essential not only for improving patient well-being but also for the comprehensive care of IBD and reducing the risk of complications. An individualized and holistic approach to EIMs is a cornerstone of successful IBD treatment [7]. This comprehensive narrative review integrates current evidence on the epidemiology, pathophysiology, diagnosis, screening, referral strategies, and treatment of EIMs across multiple organ systems. Particular emphasis is placed on practical clinical applicability, including multidisciplinary care, early recognition of referral red flags, the role of physical activity, psychological burden, and the impact of EIMs on quality of life and work productivity. By combining these domains, the review aims to provide a clinically oriented and up-to-date framework for the comprehensive management of patients with IBD and EIMs.

## 3. Epidemiology of Extraintestinal Manifestations

The prevalence and risk of developing EIMs in IBD depend on the diagnostic definitions applied. Newly established criteria developed by the European Crohn’s and Colitis Organisation (ECCO) working group in 2024 result in lower estimates of EIMs frequency. According to these guidelines, EIMs is defined as inflammatory conditions occurring outside the gut associated with IBD through immune-mediated mechanisms or shared genetic and environmental predispositions [6].

Due to variability in definitions, the reported prevalence of EIMs in IBD varies widely, ranging from 6% to 47% [4]. Furthermore, some patients experience more than one EIM. Data from the Swiss IBD Cohort Study (SIBDCS) indicate that 29% of IBD patients present with at least one EIM, and among them, 25% suffer from multiple manifestations, sometimes as many as five [5]. Most patients exhibit a single EIM (63%), while 26% have two, 5% three, 2% four, and 3% five EIMs [5].

EIMs may arise either before or after IBD diagnosis. Approximately 26% of patients experience their first EIM prior to IBD diagnosis, on average five months earlier. In the remaining 74% of cases, EIM develops after diagnosis, most commonly after approximately 92 months [3]. The most frequent manifestations occurring before the diagnosis of IBD include non-ax SpA (19.7%), (ax-SpA or ankylosing spondylitis (AS)) (39.1%), aphthous stomatitis (27.8%), and uveitis (52.2%). Less commonly reported are erythema nodosum (EN) (14.3%), pyoderma gangrenosum (PG) (14.3%), and primary sclerosing cholangitis (PSC) (23.8%) [3].

**Figure 1:**
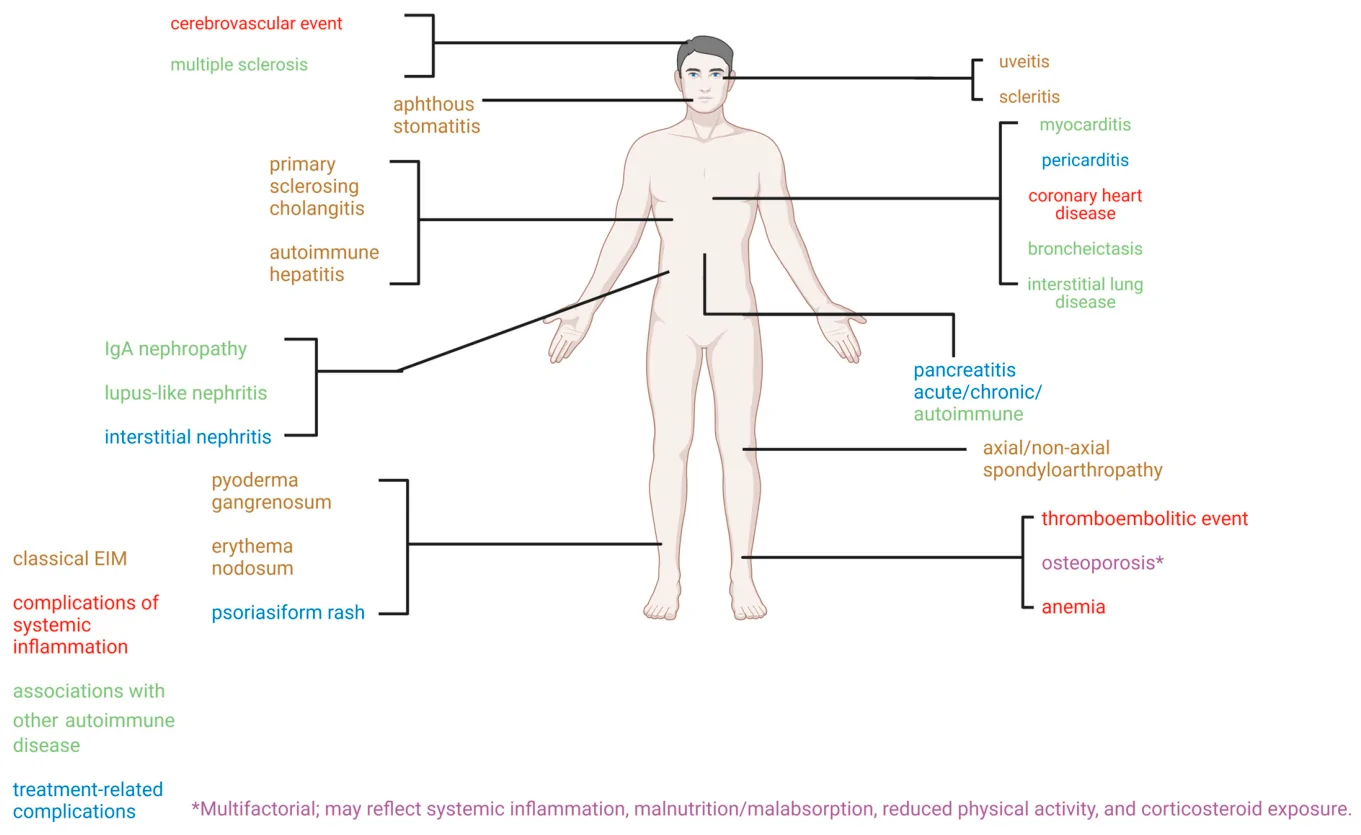
Spectrum of extraintestinal disorders associated with inflammatory bowel disease according to the 2024 ECCO framework. The figure distinguishes classical extraintestinal manifestations from associated immune-mediated disorders, complications of systemic inflammation, and treatment-related complications/adverse effects. These categories are related to IBD but are not pathophysiologically equivalent [6].

According to the 2024 ECCO framework, extraintestinal disorders associated with IBD can be broadly categorized into classical EIMs arising from inflammatory pathology at distant sites, disorders associated with other immune-mediated conditions, complications resulting from systemic inflammation, and treatment-related complications or adverse effects [6]. Importantly, these categories should not be regarded as equivalent. Classical EIMs include conditions directly linked to extraintestinal inflammatory mechanisms, whereas associated immune-mediated diseases and treatment-related adverse effects represent distinct clinical entities that may coexist with IBD but differ in pathogenesis and therapeutic implications (Figure 1) [6]:

Classical EIMs

- Uveitis, scleritis, episcleritis
- Oral manifestations
- Primary sclerosing cholangitis
- Axial and peripheral spondyloarthritis
- Pyoderma gangrenosum, erythema nodosum, Sweet syndrome

Associations with other immune-mediated disorders

- Autoimmune hepatitis
- Multiple sclerosis
- Interstitial lung disease and bronchiectasis
- Myocarditis
- Psoriasis
- Hidradenitis suppurativa

Complications of systemic inflammation

- Venous thromboembolism
- Cerebrovascular and ischaemic cardiovascular events
- Anaemia
- Fatigue
- Osteoporosis (multifactorial; may reflect systemic inflammation, malnutrition/malabsorption, reduced physical activity, and corticosteroid exposure)

Treatment-related complications/adverse effects

- Anti-TNF-induced psoriasiform reactions
- Drug-induced pancreatitis
- Drug-induced pericarditis

## 4. Pathogenesis of Extraintestinal Manifestations

The pathogenesis of EIMs in IBD remains incompletely understood, although current evidence highlights several key mechanisms. EIMs result from complex interactions between the immune system, genetic factors, and environmental influences, leading to an extended inflammatory response beyond the gastrointestinal tract [8].

Immune response transfer from the gut to other organs.

One of the primary pathophysiological mechanisms responsible for the development of EIMs in IBD is the so-called immune response transfer from the inflamed intestinal mucosa to other tissues and organs. Under physiological conditions, the gastrointestinal immune system maintains tolerance toward dietary antigens and gut microbiota. In IBD, this balance is disrupted, leading to immune activation and the production of pro-inflammatory cytokines such as Tumor Necrosis Factor alpha (TNF-α), Interleukin 12 (IL-12), IL-23 and Interferon-γ (IFN-γ) [9]. Activated T lymphocytes, particularly memory T cells, may then migrate beyond the gastrointestinal tract in response to chemokines and adhesion molecules such as MAdCAM-1 (Mucosal Addressin Cell Adhesion Molecule-1), which are expressed not only in the intestines but also in other tissues, including joints, skin, and liver [2,10]. Notably, certain extraintestinal organs express the same ligands and adhesion molecules that recruit immune cells in the intestinal wall [11]. As a result, ectopic activation of effector cells and local inflammation can occur, even in the absence of primary organ damage. This mechanism may contribute to EIMs that frequently parallel intestinal inflammation, such as erythema nodosum and some forms of peripheral SpA. However, other manifestations, particularly axial SpA and uveitis, may follow a course largely independent of intestinal disease activity [6].

### 4.1. Gut Microbiota

The role of gut microbiota in EIMs pathogenesis has not yet been clearly defined. A prevailing hypothesis suggests that molecular mimicry between gut microbiota antigens and epitopes in organs affected by EIMs may activate T lymphocyte clones and lead to cross-reactivity of the immune system; however, definitive evidence is lacking [12]. Patients with IBD and psoriatic arthritis (PsA) show decreased numbers of bacteria from the genera *Coprococcus* and *Ruminococcus*, which are essential for maintaining gut homeostasis through the production of short-chain fatty acids (SCFA) [13]. A deficiency in SCFA, known for their immunomodulatory and metabolic effects, is characteristic of these patients. Conversely, patients with IBD and SpA demonstrate increased abundance of *Dialister* species, correlating with disease activity [14]. Moreover, patients with IBD-associated arthropathy and rheumatoid arthritis (RA) show elevated levels of *Clostridiaceae*, suggesting a microbial link between different forms of arthritis [15]. Altered gut microbiota composition in IBD patients may also lead to increased expression of lipopolysaccharides (LPS), which activate pro-inflammatory molecules such as IL-6, IFN-γ, and Vascular Endothelial Growth Factor (VEGF), potentially contributing to EIMs development [16].

### 4.2. Genetic Factors

An increasing body of genetic research confirms the significant role of hereditary factors in the pathogenesis of EIMs in IBD. The occurrence of EIMs in 70% of parent–child pairs and as high as 84% in sibling pairs suggests a strong genetic component [17]. Considerable overlap has been identified in risk loci shared by IBD and EIMs, emphasizing that many immunological mechanisms driving intestinal inflammation are also involved in the development of extraintestinal changes [18].

Furthermore, association studies have linked specific gene polymorphisms (such as IL8RA, PRDM1, USP15, TIMP3) with PG, and genes like PTGER4, ITGAL, SOCS5, and CD207 with EN [19]. In patients with PSC, variants in SOCS1, STAT3, JAK2, and TYK2 have been identified—genes that are part of inflammatory pathways also associated with IBD [20].

The Arg381Gln polymorphism in the IL23R gene, known for its protective role against CD, also reduces the risk of AS, confirming the shared immunological pathways [21]. Pediatric studies have shown that patients with monogenic forms of IBD have a significantly higher prevalence of EIMs (OR = 15.36), highlighting the need for whole genome sequencing in patients with very early-onset IBD and EIMs [22].

In addition, specific human leukocyte antigen (HLA) alleles such as HLA-A2, HLA-DR1, and HLA-DQw5 in CD patients, and DRB10103, B27, and B58* in UC patients, have been linked to musculoskeletal EIM [23]. HLA-B27, found in 25–78% of IBD patients with concurrent AS, plays a crucial role in arthritis development. On the other hand, approximately 60% of AS patients present with subclinical intestinal inflammation, which may progress to overt IBD in some cases [24]. Animal models suggest that environmental triggers, such as microbiota exposure, are required for disease manifestation [25].

Identifying these genetic correlations not only enhances the understanding of EIMs pathogenesis but also paves the way for more precise risk prediction and personalized treatment strategies based on the patient’s genetic profile.

### 4.3. Mechanisms Independent of Intestinal Inflammation

Some EIMs may develop independently of active intestinal inflammation. In such cases, it is hypothesized that chronic systemic immune activation, even in the absence of overt gut inflammation, can trigger inflammatory pathways in distant organs. Persistent low-grade inflammation may lead to aberrant activation of immune cells, dysregulated cytokine release (TNF-α, IL-6, IL-17), and the formation of autoantibodies or autoreactive lymphocyte populations [26].

These mechanisms may create a pro-inflammatory microenvironment in target tissues such as the skin, eyes, joints, or hepatobiliary system, where inflammation can be initiated and sustained without a direct link to the activity of intestinal disease [3]. Additionally, genetic predisposition, shared antigens between the gut and extraintestinal tissues (molecular mimicry), and bystander activation of immune cells are believed to contribute to this independent pathogenesis [6,8,26].

**Figure 2:**
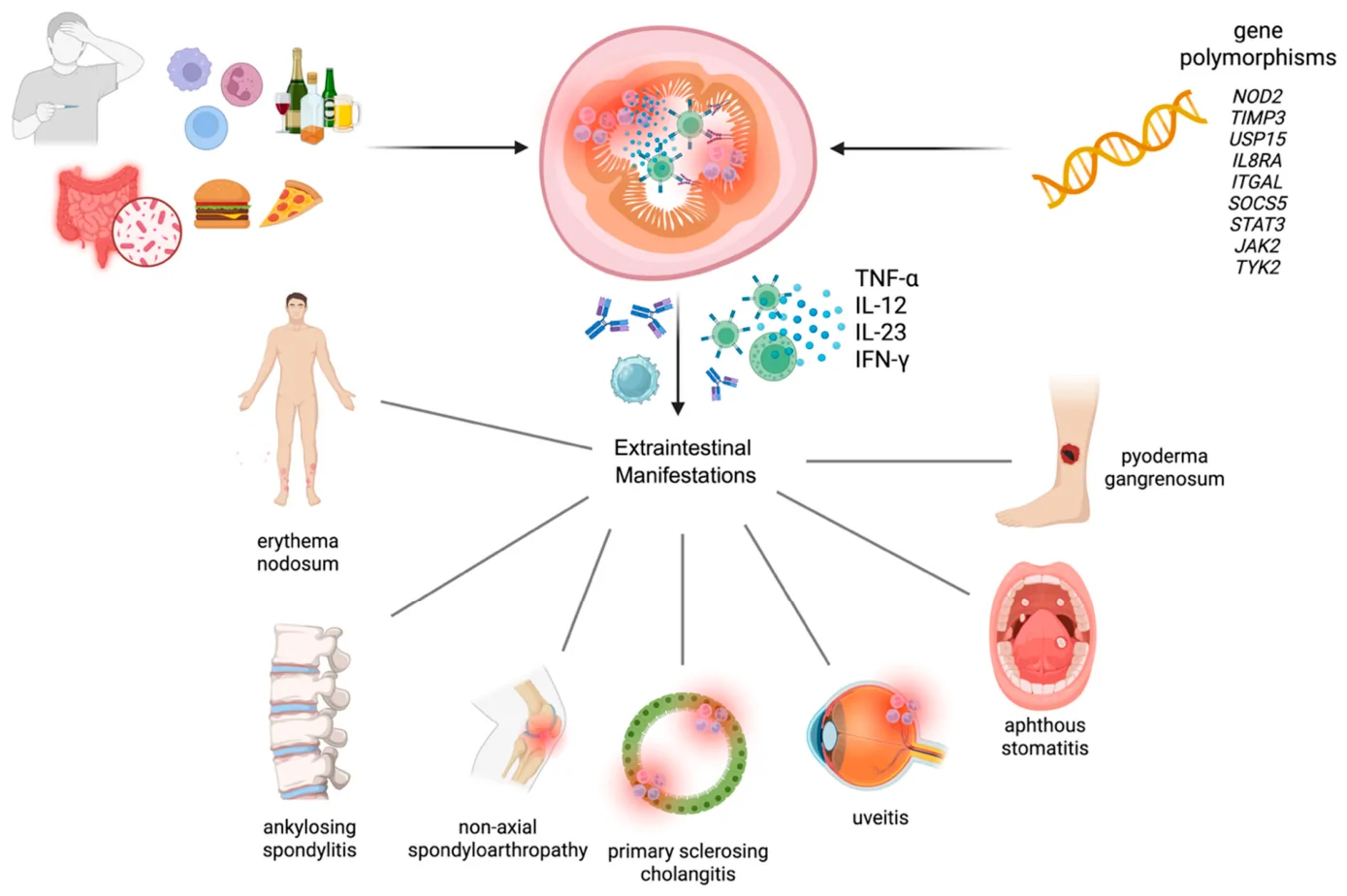
Interplay of immunological, genetic, and environmental factors in the pathogenesis of extraintestinal manifestations in inflammatory bowel disease. Created with biorender.com. IFN-γ—interpheron gamma, IL-12—interleukin 12, IL-23—interleukin 23, TNF- α—tumor necrosis factor alpha.

In summary, the pathogenesis of EIM in IBD results from the interplay of immunological, genetic, and environmental mechanisms (Figure 2). A better understanding of these processes will allow for more effective treatment of both IBD and its extraintestinal complications [8].

## 5. Clinical Manifestations of Extraintestinal Manifestations in Inflammatory Bowel Disease

### 5.1. Musculoskeletal Manifestations

SpA (spondyloarthropaties) are the most common EIMs in the course of IBD and may involve axial joints, peripheral joints, or both simultaneously, Table 1 [27]. SpA occurs with similar frequency in males and females and is more commonly observed in CD involving the colon than in UC [24]. Symptoms may occur before, concurrently with, or after the onset of gastrointestinal symptoms [28].

Ax-SpA typically presents with back pain and morning spinal stiffness, whereas the non-axial (peripheral) SpA is mainly associated with complaints in the upper and lower limbs. In terms of frequency, the peripheral form is the most common, affecting approximately 13% of patients (95% CI: 12–15%), followed by sacroiliitis (10%, 95% CI: 8–12%), with AS being the least frequent, occurring in about 3% of patients (95% CI: 2–4%) [29].

SpA is classified as a seronegative SpA due to the absence of rheumatoid factor (RF), although it is worth noting that among patients with IBD and SpA, RF positivity is observed in approximately 15%, which exceeds the prevalence in the general population (5%) [30]. Similarly, anti-citrullinated protein antibodies (ACPA) are usually negative, but occur more frequently than in the healthy population, being present in 8–12% of patients [31].

AS usually manifests with nocturnal lower back pain and morning stiffness that improves with physical activity and is often associated with uveitis [32]. Sacroiliitis, on the other hand, is frequently asymptomatic. In most patients with ax-SpA and IBD, the HLA-B27 antigen is present (25–78%), and axial joint involvement may occur independently of intestinal inflammation [33]. In CD, such changes are more common than in UC and may affect up to 25% of patients. Both AS and sacroiliitis can be detected using magnetic resonance imaging (MRI) with STIR (short tau inversion recovery) sequences [34].

Non–ax SpAs are divided into two subtypes. Type 1 is characterized by acute, asymmetric arthritis involving fewer than five joints, often large ones such as the knee. These manifestations are associated with intestinal disease activity and usually resolve spontaneously within about 10 weeks [35]. They often co-occur with other EIMs, such as EN and uveitis. Type 2 presents as symmetric arthritis involving five or more joints, typically small joints of the hands. This subtype is not associated with IBD activity and may persist long-term [36].

Among musculoskeletal EIMs in IBD patients, enthesitis, dactylitis (inflammation of the fingers), and tenosynovitis are also observed. Enthesitis may present as Achilles tendinitis, plantar fasciitis, or chest wall pain. These occur in 7–50% of adult patients with IBD, and early recognition may be facilitated by imaging studies such as ultrasonography or MRI [37].

Surgical resection of the diseased segment of the colon or total proctocolectomy in the course of UC generally leads to resolution of non-ax SpA symptoms but does not affect the course of axial joint inflammation [38]. In contrast, in CD, although colonic involvement is associated with an increased risk of non-ax SpA, surgical intervention does not appear to significantly influence its progression [39].

### 5.2. Cutaneous Manifestations

Cutaneous and mucosal EIMs represent a significant clinical issue in patients with IBD, affecting approximately 5% to 15% of individuals [40]. The most common dermal lesions include EN and PG. EN is an inflammation of the subcutaneous tissue, presenting as painful, red or violet nodules measuring 1 to 5 cm in diameter, typically located on the anterior aspect of the lower legs, but may also appear on the thighs or forearms [41]. The dermal lesions are symmetrical, non-ulcerative, and more frequently occur in females. EN is observed in approximately 5–15% of CD cases and 2–10% of UC cases [42]. Data from the SIBDCS indicate the presence of EN in both active and inactive stages of the disease, suggesting that the association between intestinal inflammation and EN may be less straightforward than previously assumed [43].

Cutaneous manifestations in IBD can be categorized into four main types based on their relationship to the underlying disease process:

1. Reactive manifestations include lesions such as EN and PG. These are thought to result from immune mechanisms active both in the gut and in the skin. They often correlate with intestinal disease activity and tend to improve with systemic treatment of IBD [43].
2. Specific manifestations refer to skin lesions that share histopathological features with intestinal inflammation. A key example is metastaic Crohn’s disease (MCD), where granulomatous lesions appear in the skin, typically in areas such as the lower limbs or genital region [44].
3. Associated manifestations are skin conditions that are more frequently seen in IBD patients but may occur independently of the intestinal inflammation. Hidradenitis suppurativa (HS) is a common example, particularly in smokers and those with higher body mass index. These conditions may reflect overlapping genetic susceptibility or environmental triggers [45].
4. Complications from treatment involve dermatologic side effects of medications used in IBD therapy. One example is the development or exacerbation of psoriasis in patients treated with TNF-α inhibitors, which may necessitate switching to an alternative agent such as ustekinumab (UST) or a Janus kinase (JAK) inhibitor (Table 2) [46].

PG is a less common but often more severe cutaneous manifestation, affecting 0.4–2.6% of IBD patients, more frequently women and individuals with colonic involvement [47]. PG usually begins as a pustule or erythematous papule that rapidly breaks down to form a deep ulcer with irregular, violet borders and a sterile purulent base [48]. The ulcers may be single or multiple, unilateral or bilateral, and can reach the size of an entire limb. PG most commonly affects the lower extremities, but lesions may also involve the head, neck, trunk, and stoma sites. Interestingly, PG can occur independently of the underlying disease activity and may even precede it. In the SIBDCS cohort, PG was documented during both active and remission phases of IBD [48].

A rare but reported dermal manifestation of IBD is Sweet’s syndrome, also known as acute neutrophilic dermatosis. It is characterized by painful nodules or plaques mainly on the limbs and trunk, often accompanied by fever and leukocytosis [49]. It is far more common in women and frequently coexists with other EIM such as arthritis or ocular symptoms [50]. The syndrome may arise before, during, or after IBD diagnosis and is occasionally associated with prior use of medications such as azathioprine (AZA) [49].

Another cutaneous EIM is MCD, in which dermal lesions exhibit histopathological features identical to those in the gastrointestinal tract, particularly non-caseating granulomas. These lesions can appear anywhere on the body but are most often localized to the genital area, including the vulva and vagina, significantly impairing patients’ quality of life [47]. Importantly, the occurrence of MCD does not correlate with intestinal inflammation activity [51].

### 5.3. Oral Manifestations

Oral manifestations are also commonly seen in patients with IBD. The most frequent are aphthous stomatitis—painful, round or oval lesions with a yellowish base and red border, usually located on the buccal or labial mucosa [52]. Their prevalence ranges from 5% to as high as 50%, occurring more often in CD and in the pediatric population. Their presence usually correlates with disease activity and with the HLA-B27 genotype [53].

Additionally, patients with IBD exhibit a higher frequency of periodontitis—a chronic inflammatory condition of the gums and surrounding periodontal tissues, leading to tooth mobility. These changes are associated with dysbiosis of the oral microbiome and external factors such as tobacco use [54].

A less common oral finding is orofacial granulomatosis (Melkersson-Rosenthal syndrome), primarily affecting young males and characterized by chronic lip swelling, gingival hypertrophy, and granulomatous changes in the skin and mucous membranes [55].

### 5.4. Ocular Manifestations

Ocular symptoms are among the more frequent, though often underrecognized, EIMs in the course of IBD, affecting between 2% and 10% of patients [56]. These manifestations can involve both the anterior and posterior segments of the eye and exhibit a diverse clinical presentation. The most common conditions include episcleritis, scleritis, and uveitis, Table 3 [57].

Episcleritis is a relatively mild and superficial ocular inflammation, typically unilateral, presenting with redness, discomfort, and mild pain. It most commonly occurs during active phases of IBD and is often associated with other EIM, such as SpAs or dermal lesions [58].

Scleritis is a more severe and deeper form of ocular inflammation, with the potential to cause complications such as globe perforation or vision loss. It is usually characterized by intense, deep ocular pain that worsens at night and radiates to the face or jaw, along with photophobia and decreased visual acuity. Although less common than episcleritis, scleritis poses a significant threat to vision and requires prompt diagnosis and management [59].

Uveitis, particularly anterior uveitis, is one of the most severe ophthalmologic EIM and may occur independently of intestinal disease activity. It frequently involves both eyes and presents with sudden onset of redness, pain, photophobia, and visual disturbances. Uveitis may develop before, concurrently with, or after the diagnosis of IBD, and in some cases, may be the initial manifestation of the underlying disease [60].

There are reports indicating a higher incidence of ocular symptoms in patients with CD compared to UC, although these differences are not unequivocal and may depend on the specific type of EIMs, age, sex, and the presence of other extraintestinal manifestations. Uveitis often coexists with ax-SpA, and the presence of the HLA-B27 antigen may increase the risk of its development.

Ocular EIM may be transient or chronic and recurrent, with the risk of permanent structural damage to the eye. Therefore, early recognition and a multidisciplinary diagnostic approach are essential, particularly given that these symptoms do not always correlate with intestinal disease activity [58].

### 5.5. Hepatobiliary Manifestations

Hepatobiliary manifestations represent a significant, yet often underrecognized group of EIMs in patients with IBD, Table 4. It is estimated that up to 20–40% of IBD patients may present with abnormal liver function tests during the course of the disease [61]. Among these, the most frequently diagnosed condition is PSC, which is closely associated with UC. Approximately 70–80% of patients with PSC have coexisting UC, although PSC can also occur in patients with CD, particularly those with colonic involvement [62].

PSC is a chronic, progressive, cholestatic inflammatory disease that leads to fibrosis and strictures of both intrahepatic and extrahepatic bile ducts. The clinical presentation of PSC may be mild or nonspecific, and in most cases, the disease is diagnosed based on abnormal liver enzyme tests, primarily elevated alkaline phosphatase levels. Interestingly, PSC can be diagnosed independently of IBD activity and may even precede the onset of intestinal symptoms. Over time, PSC may lead to liver cirrhosis, portal hypertension, and secondary biliary hepatopathy [61].

A characteristic feature of PSC is its association with an increased risk of developing cholangiocarcinoma, the incidence of which is markedly higher than in the general population. Furthermore, in patients with both IBD and PSC, the risk of colorectal cancer is increased sevenfold. Therefore, colonoscopic surveillance is recommended once a year for individuals with concomitant PSC, regardless of IBD activity [63].

AIH is a chronic inflammatory liver disease characterized by immune-mediated destruction of hepatocytes. It is associated with the presence of autoantibodies, elevated serum transaminases, and characteristic histopathological findings such as interface hepatitis. Although less common in patients with IBD, AIH may coexist with both CD and UC, contributing to hepatobiliary involvement. Diagnosis requires exclusion of other liver pathologies and is confirmed through a combination of serologic, biochemical, and histological criteria. Immunosuppressive therapy, typically with corticosteroids and AZA, remains the mainstay of treatment [64].

Other liver disorders associated with IBD include metabolic dysfunction-associated steatotic liver disease (MASLD), which can occur in both active and inactive phases of IBD [6]. The development of MASLD in these patients is likely multifactorial. Potential contributing factors include chronic systemic inflammation promoting insulin resistance, alterations in gut microbiota and increased intestinal permeability leading to metabolic endotoxemia, side effects of long-term corticosteroid or immunosuppressive therapy, and lifestyle-related factors such as reduced physical activity during disease flares and dietary imbalances [6]. Additionally, overlapping components of metabolic syndrome—such as obesity, dyslipidemia, and type 2 diabetes—may further increase the risk of MASLD in individuals with IBD.

### 5.6. Other Health Burdens Among Patients with IBD

Patients with IBD have a significantly increased risk of VTE, primarily involving deep vein thrombosis of the lower extremities and pulmonary embolism. Numerous studies have demonstrated that the risk of these events is 2–3 times higher in individuals with IBD than in the general population [65]. VTE can occur during disease flares as well as in remission, with the highest risk observed during hospitalization and postoperative periods. The underlying pathophysiological mechanisms include chronic inflammation, endothelial dysfunction, hypercoagulability, and hemodynamic disturbances [66]. Additional risk factors include immobilization, dehydration, nutritional deficiencies, and the use of certain medications (e.g., corticosteroids). Thrombosis may also involve splanchnic vessels, such as the portal, mesenteric, and splenic veins, leading to serious and often diagnostically challenging complications. Due to their asymptomatic or nonspecific presentation, thromboembolic events in IBD may go undiagnosed, highlighting the need for a high degree of clinical vigilance.

Anemia is one of the most common hallmarks of IBD affecting 20–70% of patients depending on disease activity and population studied. It results from multiple factors, the most important being chronic gastrointestinal blood loss, iron deficiency, chronic inflammation, and deficiencies of vitamin B12 and folic acid. Anemia may constitute a first sign suggesting underlying IBD before typical gastrointestinal symptoms will develop [67]. Iron deficiency anemia is the most prevalent form and often coexists with anemia of chronic disease (ACD) [68]. Persistent inflammation increases hepcidin production, which reduces intestinal iron absorption and its availability in the bone marrow. Vitamin B12 deficiency is especially common in CD patients with involvement of the terminal ileum, while folic acid deficiency may arise from malabsorption or drug effects, such as those associated with sulfasalazine [69]. Anemia in IBD negatively impacts quality of life, reducing physical performance, concentration, and mental well-being. Importantly, it may occur independently of intestinal inflammation and should be regularly monitored in all IBD patients.

Rare renal EIM include immune-mediated nephropathies such as IgA nephropathy (the most common glomerular disease associated with IBD), lupus-like nephritis, and interstitial nephritis. Renal manifestations may result directly from active inflammation or secondarily from treatment-related toxicity, such as that caused by immunosuppressive agents. Nephrolithiasis and secondary renal amyloidosis may also occur in the course of IBD [70]. A notable recent development in the early detection of chronic kidney disease (CKD) is the use of the albumin-to-creatinine ratio (ACR). This simple, non-invasive test allows for the detection of early kidney damage before a decline in glomerular filtration rate becomes apparent. In patients with IBD, ACR measurement may be useful for identifying subclinical renal involvement, enabling earlier intervention and monitoring. According to KDIGO guidelines, an ACR value greater than 30 mg/g (3 mg/mmol), confirmed in at least two measurements taken over a period of three months or more, warrants the diagnosis of CKD stage G1 when glomerular filtration rate is ≥90 mL/min/1.73 m^2^ [68]. However, the role of ACR in detecting kidney involvement specifically in IBD patients has not yet been fully established and requires further large-scale, prospective studies [71].

The respiratory system may also be involved in the inflammatory process. Cases have been reported of bronchitis, tracheitis, chronic cough, and pulmonary infiltrates that may resemble infectious or autoimmune diseases. Pulmonary EIM can occur independently of intestinal disease activity and sometimes pose a diagnostic challenge [72].

Cardiovascular involvement in IBD is rare, but pericarditis, myocarditis, and non-cardiogenic pulmonary edema have been described [6]. Additionally, the chronic systemic inflammation associated with IBD may increase the risk of atherosclerosis and cardiovascular events.

Neurological symptoms are among the rarest but potentially most concerning EIMs. Cases have been reported of peripheral neuropathy, optic neuritis, inflammatory myopathies, and central nervous system demyelination, which can clinically mimic multiple sclerosis. In some instances, neurological symptoms may result from medication toxicity or nutritional deficiencies (e.g., vitamin B12) related to IBD [6].

Although these less common EIMs occur less frequently than the classical forms, their presence can significantly affect patient QoL. Their multisystemic nature requires diagnostic vigilance and the involvement of a multidisciplinary team.

### 5.7. Practical Screening and Referral for Extraintestinal Manifestations

Systematic screening for extraintestinal manifestations should be incorporated into routine follow-up of patients with IBD, including during intestinal remission, because several EIMs may evolve independently of bowel disease activity (Table 5). Clinicians should actively inquire about inflammatory musculoskeletal symptoms, new skin lesions, ocular pain or visual disturbances, and hepatobiliary abnormalities [6]. Selected screening tools may facilitate early recognition, particularly for spondyloarthritis (Table 6). The DETAIL questionnaire was developed specifically to screen patients with IBD for features suggestive of spondyloarthritis [73], while the IBIS-Q was designed to detect both axial and peripheral SpA in this population [74]. Referral to rheumatology should be considered in the presence of chronic inflammatory back pain, peripheral joint swelling, dactylitis, or enthesitis, which have been identified as relevant referral “red flags” in patients with IBD [75]. For ocular manifestations, the OMIS questionnaire has been proposed as a screening tool to support the identification of patients requiring ophthalmological assessment [76]. These instruments should support, rather than replace, clinical assessment, and no single validated questionnaire currently covers the full spectrum of EIMs.

## 6. Treatment of Extraintestinal Manifestations

The management of classical EIMs in IBD remains challenging because individual manifestations differ in their relationship with intestinal disease activity and in the quality of available therapeutic evidence. In the following section, evidence for classical IBD-related EIMs is presented separately from data derived from associated immune-mediated disorders or therapeutic indications outside IBD. Evidence extrapolated from other immune-mediated diseases is considered indirect and should not be interpreted as proof of efficacy for IBD-associated EIMs.

Among systemic therapies, TNF-α antagonists have the most consistent evidence across several classical EIMs, particularly musculoskeletal manifestations and pyoderma gangrenosum [6]. Evidence for newer biologics and small molecules is more heterogeneous and frequently derives from observational studies, post hoc analyses, case series, or studies conducted in non-IBD populations [6]. Vedolizumab, owing to its gut-selective mechanism, has shown limited or inconsistent efficacy for EIMs that follow a course independent of intestinal inflammation, particularly axial SpA [6,77].

Emerging therapies, including JAK and IL-23 inhibitors, may offer additional therapeutic opportunities; however, their efficacy should be considered separately for each EIM. For example, JAK inhibitors have demonstrated efficacy in axial SpA populations without IBD, but dedicated evidence in IBD-associated axial SpA remains limited [6,78]. Similarly, EIM-specific data for IL-23 inhibitors remain insufficient. Post hoc analyses, including data from the VIVID-1 programme, suggest a potential effect of mirikizumab on selected EIMs, but these findings require confirmation in prospective studies with predefined EIM-specific endpoints [78].

Accordingly, therapeutic decisions should consider both intestinal disease activity and the specific EIM phenotype, with preference for agents supported by direct evidence whenever available (Table 7). Evidence derived exclusively from associated immune-mediated conditions such as psoriasis, psoriatic arthritis, or hidradenitis suppurativa is discussed separately and is not considered direct evidence for classical IBD-related EIMs.

The 2024 ECCO guidelines emphasize that some EIMs (PA, EN) often improve with control of intestinal inflammation. Consequently, gut-selective therapies such as VDZ, etrasimod (ETRA), or OZA, while not specifically developed for EIMs, may indirectly alleviate these manifestations through their ability to reduce mucosal inflammation and induce remission in IBD. However, for EIMs that are independent of intestinal activity (ax SpA, uveitis, or PSC), these agents are unlikely to be effective [6].

Currently, European Medicines Agency (EMA) and Food and Drug Administration (FDA) approvals for UC include anti-TNF agents (IFX, ADA, GOL), VDZ, UST, IL-23 inhibitors (GUS, RISA, MIRI), S1P modulator (ETRA, OZA), and JAK inhibitors (TOFA, FILGO, UPA). In Crohn’s disease, EMA has approved IFX, ADA, GOL, VDZ, UST, RISA, and UPA, with FDA additionally authorizing GUS and MIRI.

### 6.1. Axial Spondyloarthropathy (ax-SpA)

Management of axial SpA in patients with IBD requires simultaneous consideration of intestinal and musculoskeletal disease. Physiotherapy and regular exercise represent important components of management. NSAIDs can reduce spinal pain and stiffness but should be used cautiously, particularly in patients with active IBD, because of the potential risk of exacerbating intestinal disease [6,79].

TNF-α antagonists are the preferred biologic therapy for patients with active IBD-associated axial SpA requiring systemic treatment [6]. Although no randomized controlled trial has specifically evaluated biologic therapy for axial SpA in an IBD population, four open-label studies including approximately 100 patients demonstrated improvement in SpA symptoms with TNF-α antagonists [6,80]. Etanercept should be avoided when treatment of both conditions is required because it lacks efficacy for intestinal IBD and has been associated with paradoxical gastrointestinal inflammation [6,81].

Evidence for ustekinumab and vedolizumab in IBD-associated axial SpA remains inconsistent. Available studies are predominantly retrospective, frequently combine axial and peripheral manifestations, and provide insufficient evidence to support routine use of either drug for axial disease. Accordingly, the 2024 ECCO guidelines do not recommend vedolizumab or ustekinumab for the treatment of IBD-associated axial SpA [6].

JAK inhibitors represent a potentially relevant therapeutic option because some agents are effective in both IBD and axial SpA. However, evidence for axial efficacy is derived predominantly from trials conducted in patients with axial SpA without concomitant IBD [82]. Dedicated trials in IBD-associated axial SpA are lacking; therefore, their role in this setting should currently be regarded as supported by indirect evidence and pharmacological rationale rather than EIM-specific randomized evidence [6].

### 6.2. Non–Axial (Peripheral) Spondyloarthropathy (Non–ax SpA)

Evidence for the treatment of IBD-associated peripheral SpA is also limited by the absence of dedicated randomized controlled trials. Available evidence derives predominantly from open-label studies, observational cohorts, and post hoc analyses [6]. Control of intestinal inflammation may improve peripheral arthritis, particularly manifestations that parallel IBD activity.

TNF-α antagonists have the strongest supportive evidence and are recommended by ECCO for IBD-associated non-axial SpA [6]. A pooled post hoc analysis of adalimumab trials demonstrated significantly greater resolution of arthritis and arthralgia compared with placebo, while several open-label studies have reported improvement in peripheral joint manifestations with TNF-α antagonists [6].

Methotrexate and sulfasalazine may be considered for selected peripheral manifestations, particularly when intestinal disease does not itself require advanced therapy. Ustekinumab may also provide benefit in peripheral SpA, although the evidence remains less robust than for TNF-α antagonists [6].

Evidence regarding JAK inhibitors specifically in IBD-associated peripheral SpA remains limited. Their established efficacy in other inflammatory arthritides should be regarded as indirect supportive evidence and should not be extrapolated as proof of efficacy in IBD-associated peripheral SpA. Treatment decisions should therefore be individualized in collaboration between gastroenterologists and rheumatologists [6].

### 6.3. Treatment of Cutaneous Manifestations

Management of cutaneous manifestations in IBD depends on their classification, severity, and relationship with intestinal disease activity. Classical cutaneous EIMs, including erythema nodosum (EN) and pyoderma gangrenosum (PG), should be distinguished from IBD-associated immune-mediated skin diseases such as psoriasis and hidradenitis suppurativa (HS), as well as from treatment-induced dermatological adverse effects [6].

EN frequently parallels intestinal disease activity and management primarily focuses on adequate control of the underlying IBD. Severe or persistent manifestations may require a short course of systemic corticosteroids, while TNF-α antagonists can be considered in refractory cases or when treatment is also required for active intestinal disease [6,75].

PG may occur independently of intestinal disease activity and often requires specific systemic treatment. Systemic corticosteroids and ciclosporin are established therapeutic options, while TNF-α antagonists, particularly infliximab, have the strongest biologic evidence and may be considered in moderate-to-severe or refractory disease [6,79]. Other agents, including ustekinumab, have been used in refractory cases, although the supporting evidence remains limited. Early dermatological involvement is recommended.

Sweet syndrome usually responds to systemic corticosteroids, while metastatic Crohn’s disease requires individualized treatment and may respond to topical or systemic corticosteroids, antibiotics, immunomodulators, or TNF-α antagonists [74].

Psoriasis and HS should be considered IBD-associated immune-mediated skin disorders rather than classical reactive EIMs. Their management should therefore follow disease-specific recommendations while taking concomitant IBD into account [6]. Adalimumab remains an established therapeutic option for HS; however, emerging evidence suggests that JAK inhibitors may also have therapeutic potential in this setting. Available data are currently limited mainly to case reports and small case series, including reports of successful JAK inhibitor use in patients with concomitant IBD and hidradenitis suppurativa, and should therefore be interpreted cautiously [83].

Treatment-induced psoriasiform reactions, particularly those associated with TNF-α antagonists, should likewise be distinguished from pre-existing or IBD-associated psoriasis. Management depends on severity and may include topical therapy, continuation of TNF-α inhibition in mild cases, or switching to an alternative therapeutic class in severe or refractory disease [6].

### 6.4. Treatment of Ocular Complications

Management of ocular EIMs depends on the specific diagnosis, severity, and relationship with intestinal disease activity. Mild episcleritis frequently parallels active IBD and may improve with treatment of the underlying intestinal inflammation. Supportive therapy with artificial tears or cold compresses may be sufficient, while topical anti-inflammatory treatment can be considered in persistent cases.

Uveitis requires urgent ophthalmological assessment because untreated inflammation may result in permanent visual impairment. Topical corticosteroids are recommended as first-line therapy for anterior uveitis. Intermediate, posterior, or panuveitis may require intravitreal or systemic corticosteroids, with steroid-sparing immunomodulators such as methotrexate or mycophenolate mofetil in selected patients [6].

In persistent or sight-threatening uveitis, particularly when active IBD coexists, adalimumab or infliximab may be considered [84]. Importantly, evidence for non-TNF biologics and small molecules in IBD-associated uveitis remains insufficient, and the 2024 ECCO guidelines note the absence of trial evidence supporting their efficacy in this specific setting [6].

Scleritis also requires prompt ophthalmological involvement and may necessitate systemic anti-inflammatory or immunosuppressive treatment according to severity [6].

### 6.5. Treatment of Hepatobiliary Disease

Management of hepatobiliary disorders associated with IBD depends on the specific diagnosis and should distinguish classical hepatobiliary EIMs from associated immune-mediated liver disease and treatment-related hepatotoxicity.

For PSC, no pharmacological treatment has convincingly been shown to halt disease progression. Ursodeoxycholic acid may improve biochemical markers in selected patients but has not demonstrated a clear survival benefit or reduction in cholangiocarcinoma risk [85]. Management therefore focuses on surveillance, treatment of complications, and endoscopic management of clinically relevant biliary strictures. In advanced disease or liver failure, liver transplantation remains the definitive therapeutic option [59,85,86].

Biologic and small-molecule therapies used for IBD should be selected primarily according to intestinal disease activity, as there is currently insufficient evidence that they modify the natural history of concomitant PSC [6,87].

Autoimmune hepatitis, including PSC–AIH overlap syndromes, should be distinguished from PSC and managed according to disease-specific hepatological recommendations. Corticosteroids, with or without azathioprine, remain the standard immunosuppressive approach for AIH [88].

**Figure 3:**
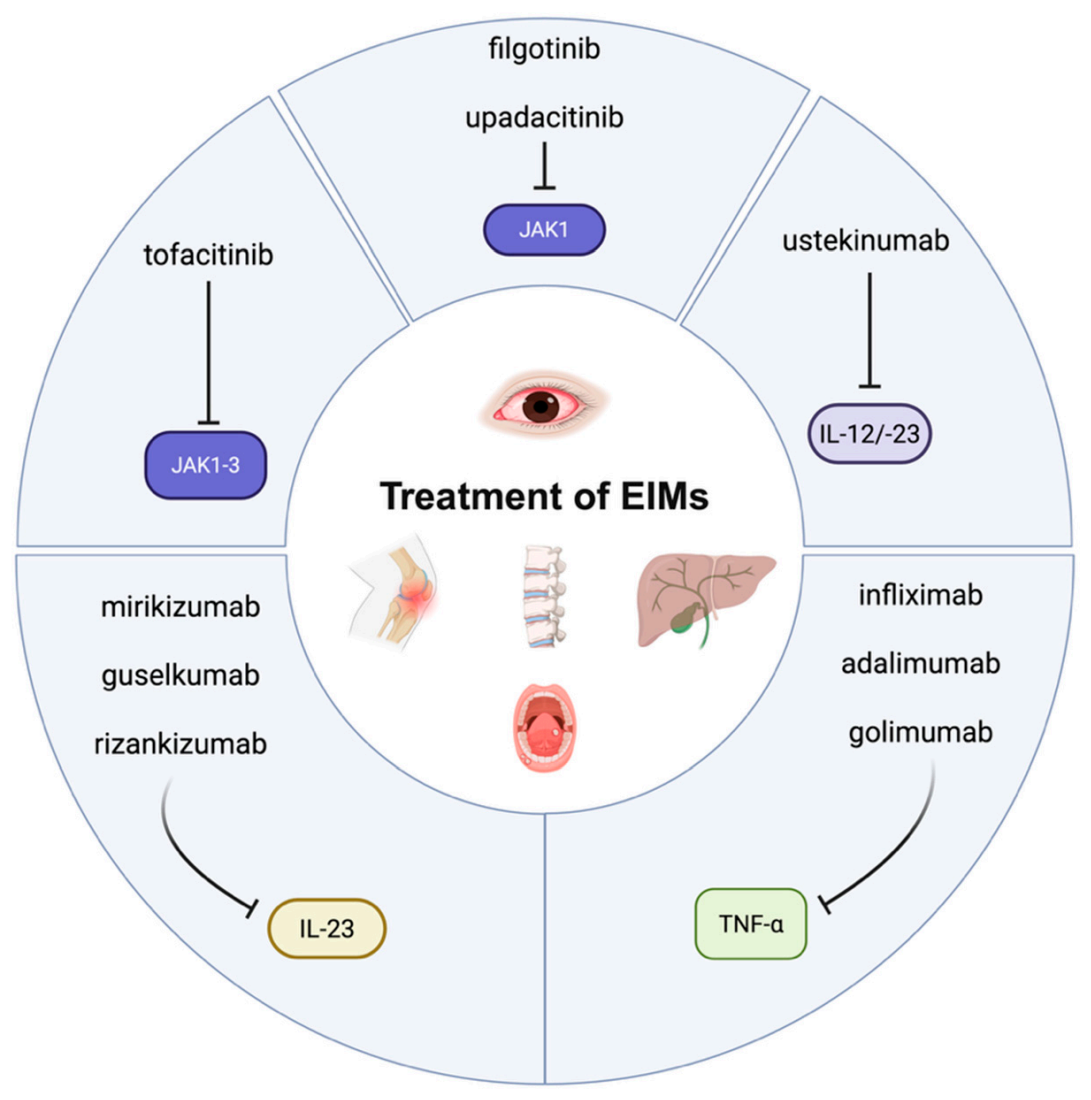
The overview of biological/small molecule treatment targets in the therapy of the selected extraintestinal manifestations. EIM—extraintestinal manifestations, IL-12—interleukin 12, IL-23—interleukin 23, JAK—Janus kinase, TNF-a—tumor necrosis factor alpha.

Other hepatobiliary abnormalities, including metabolic dysfunction-associated steatotic liver disease and drug-induced liver injury, should not be considered equivalent to classical hepatobiliary EIMs. Their management should target the underlying metabolic or treatment-related cause (Figure 3).

The strongest evidence in treatment of EIMs supports systemic agents (anti-TNFα and JAK inhibitors), which show efficacy across a broad spectrum of symptoms. In contrast, gut-selective therapies such as VDZ or OZA have limited or mixed effects. Further dedicated trials with EIM as primary endpoints are needed to optimize treatment strategies.

**Summary**

Analysis of the diverse EIMs in IBD—including musculoskeletal, dermatologic, ocular, hepatobiliary, hematologic, thromboembolic, and other systemic complications—confirms their high frequency and substantial clinical impact. EIMs are estimated to occur in 25–47% of IBD patients and may significantly affect disease burden, morbidity, and long-term prognosis [8,10,18]. Certain EIMs, such as non-ax SpA, aphthous stomatitis, or EN, typically correlate with intestinal disease activity and often resolve with adequate control of gut inflammation [13,14,29]. In contrast, others—including uveitis, ax-SpA, and PSC—commonly follow an independent clinical course and may arise even in the absence of gastrointestinal symptoms [15,17,18,29].

**Figure 4:**
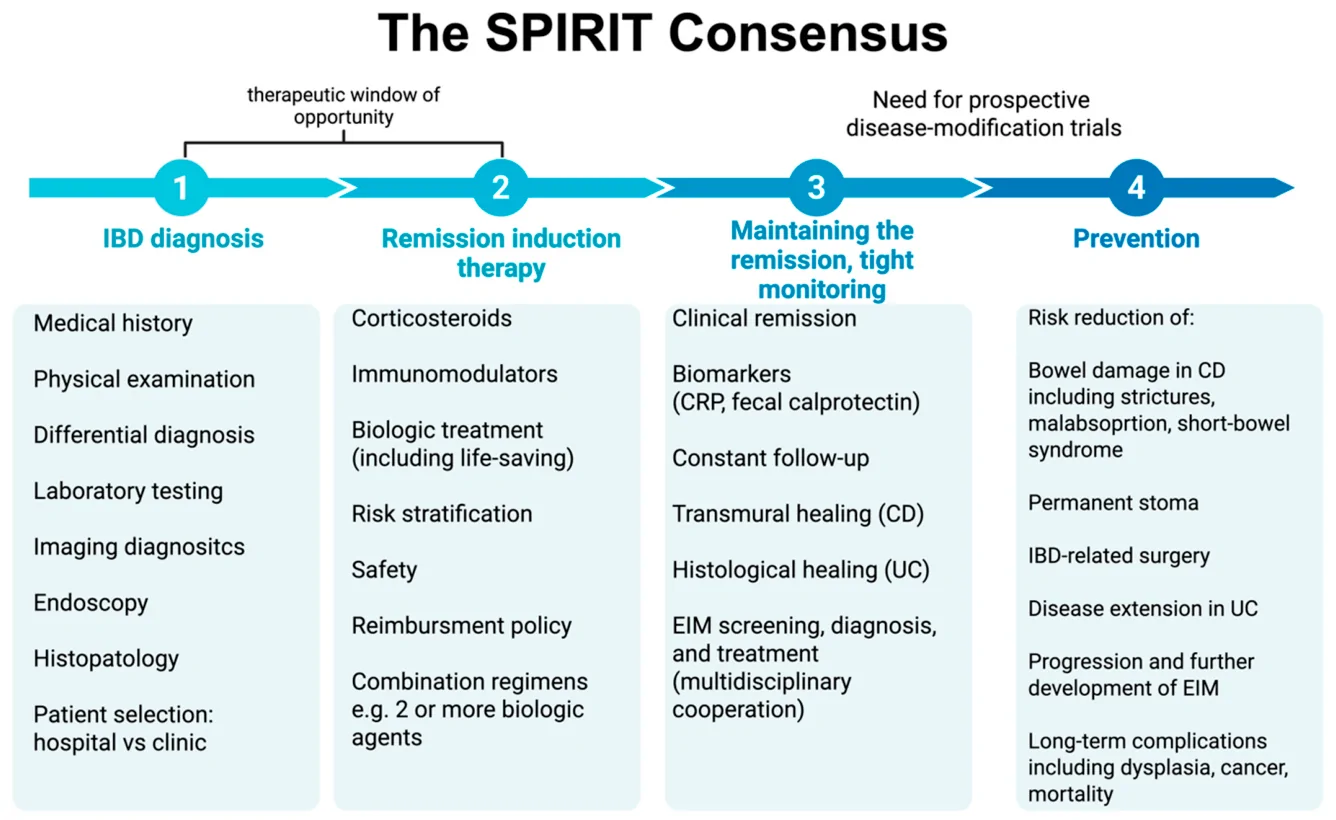
Assumptions of the SPIRIT consensus in selecting endpoints for inflammatory bowel disease modification trials with special emphasis on achieving ultimate treatment goals, namely, histological healing in ulcerative colitis and transmural healing in Crohn’s disease. CD—Crohn’s disease. CRP—C-reactive protein. UC—ulcerative colitis.

Contemporary treatment of IBD is based on a treat-to-target strategy, in accordance with the Selecting Therapeutic Targets in Inflammatory Bowel Disease II (STRIDE II) consensus guidelines [89]. These guidelines define therapeutic goals that include clinical response, clinical remission, normalization of inflammatory biomarkers (C-reactive protein and fecal calprotectin), and endoscopic healing of mucosal inflammation. A novel approach to IBD management is outlined in the Selecting End Points for Disease-ModIfication Trials (SPIRIT) [90] consensus, which emphasizes long-term treatment optimization through improvements in QoL, reduction in disability risk, prevention of complications (including EIMs), minimization of oncologic risk, and mortality reduction. However, implementation of the SPIRIT consensus in clinical practice remains a matter of debate due to the lack of high-quality prospective studies assessing its effect on disease course modification. In the era of evidence-based medicine, further data are warranted to validate the efficacy of this approach. The newly proposed SPIRIT treatment targets include early diagnosis of IBD, prompt initiation of therapy to prevent stricture formation, enhanced monitoring of therapeutic response, and optimization of drug selection, including combination regimens. All these aspects may help to achieve ultimate treatment goals namely, histological healing for UC and transmural healing for CD (Figure 4). There is an increasing tendency to evaluate treatment efficacy not only in terms of intestinal symptom remission but also in achieving remission of EIMs [89,90].

### 6.6. Physical Activity as Part of Multidisciplinary Care

Physical activity should be considered an integral component of multidisciplinary IBD care, with potential benefits for physical fitness, body composition, cardiovascular health, and musculoskeletal symptoms, although the quality of evidence regarding its effect on intestinal disease activity remains heterogeneous. The BE-FIT-IBD study showed that physical inactivity is common among patients with IBD, with 42.9% of participants classified as inactive and only 4.1% achieving health-enhancing levels of physical activity; importantly, fear of disease exacerbation and IBD-related limitations were frequently reported barriers [91]. A post hoc analysis further demonstrated that social context may influence physical activity: lack of support from relatives or friends was associated with more negative beliefs about exercise, while partner status and the attitudes of the social network affected perceived barriers to regular activity [92]. The BE-FIT-IBD-2 study additionally identified age ≥50 years as an independent predictor of physical inactivity, with inactive behavior reported in 59.1% of patients aged ≥50 years compared with 39.6% of younger patients (OR 3.30, 95% CI 1.22–8.91) [93]. These findings suggest that counselling regarding physical activity should be individualized and should consider age, disease-related barriers, and the patient’s social environment.

### 6.7. Psychological and Psychiatric Burden in IBD

Psychological and psychiatric comorbidities are common in patients with IBD and represent an important component of the overall systemic disease burden. A systematic review and meta-analysis by Barberio et al. showed that symptoms of anxiety affect approximately one-third of patients with IBD, whereas depressive symptoms occur in approximately one-quarter, with both being substantially more frequent in patients with active disease [94]. The relationship between psychological symptoms and intestinal disease appears to be bidirectional; longitudinal evidence suggests that anxiety and depression may be associated with adverse IBD outcomes, while active intestinal disease increases the subsequent risk of psychological symptoms [95]. These comorbidities may adversely affect quality of life, social functioning, treatment adherence, and patients’ perception of disease burden. Psychological assessment and timely access to psychological or psychiatric support should therefore form part of multidisciplinary IBD care. Psychological interventions probably provide small beneficial effects on quality of life and symptoms of anxiety and depression, although their effect on intestinal inflammation remains uncertain [96].

### 6.8. Quality of Life and Work Productivity

Beyond their direct clinical consequences, IBD and its EIMs may substantially impair patients’ quality of life, daily functioning, and work productivity. Musculoskeletal symptoms, fatigue, pain, psychological distress, and active intestinal disease can contribute to absenteeism and reduced performance at work. The IBD-GO-WORK study, including 300 patients with IBD, demonstrated a substantial occupational burden, with a median of 56 h of absolute absenteeism over four weeks; moreover, 75.3% of participants reported at least 25% loss of work productivity [97]. Disease activity correlated strongly with absenteeism, while anxiety was independently associated with reduced work productivity, highlighting the interplay between intestinal, psychological, and functional disease burden [97].

## 7. Future Perspective on Management of Extraintestinal Manifestations of Inflammatory Bowel Disease

The pathogenesis of EIM is complex and multifactorial, involving shared immune mechanisms, dysregulated lymphocyte trafficking, genetic predisposition, and gut microbiota dysbiosis [13,14,17,22]. Immunologic mechanisms include the migration of activated T cells from the intestinal mucosa to extraintestinal sites via shared adhesion molecules such as MAdCAM-1 [13]. Genetic studies have demonstrated a significant overlap of susceptibility loci for IBD and EIM, including polymorphisms in NOD2, IL23R, and HLA alleles such as HLA-B27 [22,24]. Additionally, gut microbial imbalance contributes to immune dysregulation through altered production of SCFAs and increased expression of pro-inflammatory mediators such as IL-6 and VEGF [14,15].

Biologic therapies—particularly TNF-α inhibitors such as IFX and ADA have significantly advanced the management of EIM. These agents have demonstrated efficacy in reducing articular and ocular inflammation, and are considered first-line treatments for many moderate to severe manifestations [6,79,84]. However, paradoxical worsening of EIMs has been observed with gut-selective agents such as VDZ, emphasizing the potential for distinct immunopathological pathways between intestinal and extraintestinal inflammation [77]. Clinical observations further reveal that EIM may develop even during effective intestinal therapy, reinforcing the need for a comprehensive, integrated approach to care. The heterogeneity of EIM presentations and their asynchronous relationship with intestinal disease necessitate tailored diagnostic and therapeutic strategies. Despite therapeutic advances, data on the efficacy of newer biologics and small-molecule therapies in specific EIM subtypes remain limited [82]. Most available evidence arises from retrospective studies, post hoc analyses, or small case series. There is a pressing need for robust, prospective clinical trials specifically addressing EIM as primary endpoints.

Key priorities for future research should include:

- Designing randomized controlled trials with clearly defined EIM-specific outcomes.
- Developing predictive biomarkers to identify patients at risk of EIM and to guide individualized therapy.
- Investigating the role of combination therapies (e.g., biologics with JAK inhibitors) in managing refractory EIM.
- Incorporating EIM evaluation into future drug approval protocols for IBD therapeutics.

Such efforts are essential to deepen the understanding of EIM pathophysiology and to enable precise, effective treatment selection.

**Figure 5:**
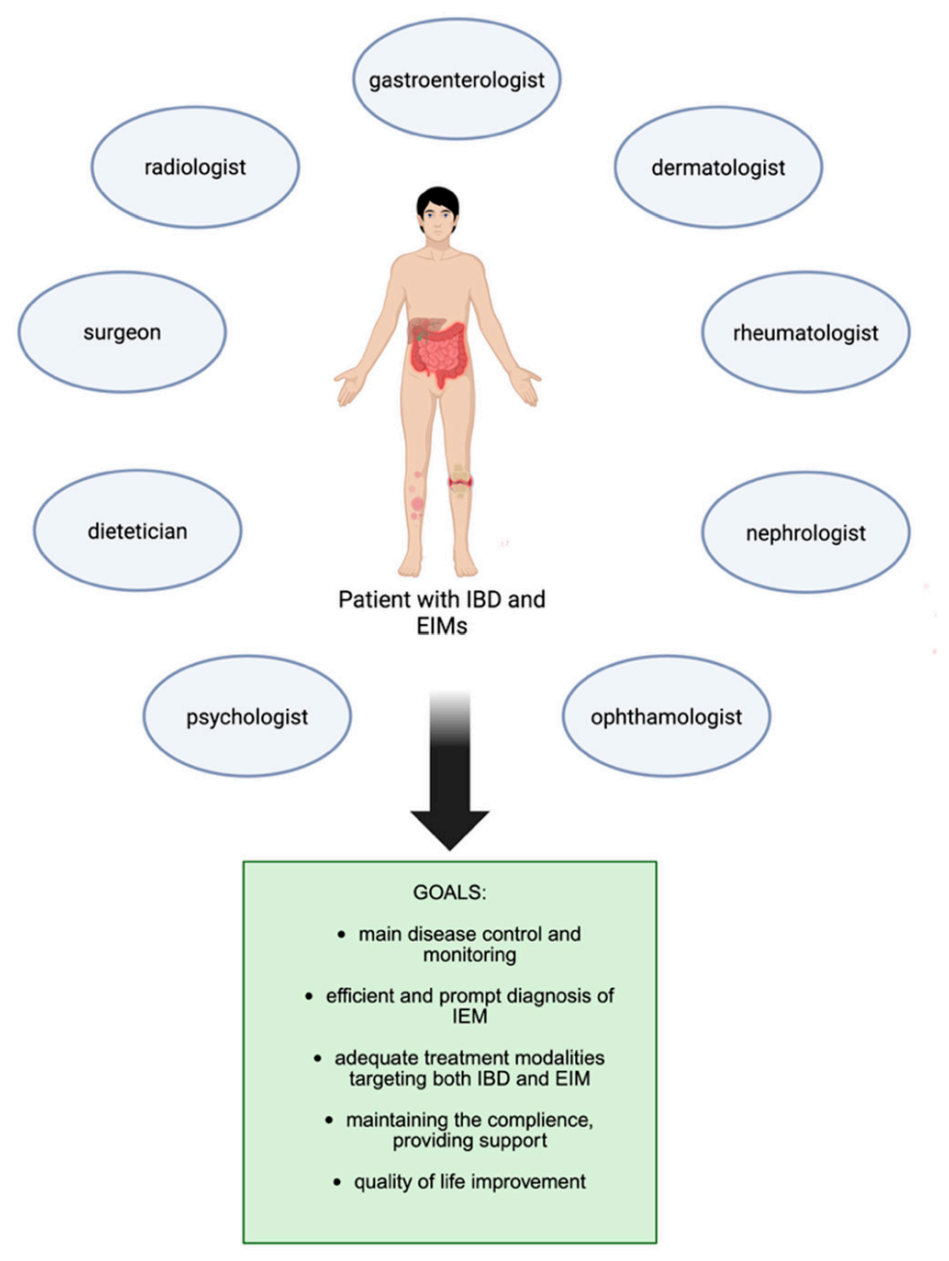
A multidisciplinary, patient-centered approach towards management of EIM during course of the inflammatory bowel disease. Created with biorender.com. IBD—inflammatory bowel disease, EIM—extraintestinal manifestations.

Optimal management of EIMs in IBD necessitates a coordinated, multidisciplinary approach (Figure 5). Effective care requires the collaboration of gastroenterologists with rheumatologists, dermatologists, ophthalmologists, hepatologists, nephrologists, pulmonologists, psychiatrist, and neurologists [98]. This is especially critical given the multisystemic nature of EIMs and their potential to cause irreversible organ damage if unrecognized or undertreated. Timely inter-specialty communication is essential for accurate diagnosis, comprehensive monitoring, and timely adjustment of therapy. Integration of multidisciplinary expertise ensures that both intestinal and extraintestinal disease burdens are addressed, leading to improved clinical outcomes and patient QoL.

Establishing structured, multidisciplinary IBD clinics or care pathways can significantly enhance therapeutic efficiency, facilitate early detection of EIM, and optimize use of advanced treatment modalities [98]. Additionally, such models support shared decision-making, personalized treatment planning, and improved patient education and adherence. The involvement of allied health professionals, including dietitians, psychologists, and specialist nurses, further strengthens this holistic model of care.

## 8. Conclusions

EIMs represent a critical component of IBD that requires vigilant recognition and targeted intervention. While therapeutic progress has been achieved, there remains a substantial gap in high-quality evidence for managing many EIM subtypes. A multidisciplinary, patient-centered strategy is therefore not only beneficial but essential for achieving optimal disease control and long-term outcomes in individuals with IBD. As our understanding of EIM continues to evolve, so too must our clinical practices—embracing collaboration, innovation, and research-driven care to meet the complex needs of this population. Despite the increasing number of therapeutic options for IBD, the evidence supporting the management of EIMs remains heterogeneous. The strongest evidence is available for anti-TNF therapy, whereas data for several newer biologics and small molecules are derived predominantly from post hoc analyses, observational studies, and small case series. Moreover, EIMs are rarely included as predefined primary endpoints in IBD clinical trials, limiting direct comparisons between therapeutic strategies. Evidence is also uneven across organ systems, with substantially more data available for musculoskeletal and dermatological manifestations than for ocular, hepatobiliary, renal, or pulmonary involvement. Consequently, current therapeutic decisions frequently require integration of IBD-specific evidence with data extrapolated from rheumatology, dermatology, and other specialties. Future prospective studies should therefore incorporate standardized EIM definitions and EIM-specific outcomes to provide a more robust basis for treatment recommendations.

## Figures and Tables

**Table 1:** Prevalence, clinical features, and diagnostic tools for SpAs subtypes in patients with IBD. IBD—inflammatory bowel diseases, MRI—magnetic resonance imaging, STIR—short tau inversion recovery (MRI sequence).

Condition	Prevalence	Clinical Features	Diagnostic Tools
Axial spondyloarthritis (ax-SpA)	Not specified separately	Back pain, morning stiffness, associated with uveitis, independent of IBD activity	MRI (STIR sequences), clinical evaluation
Non-axial (peripheral) Spondyloarthritis (non-ax SpA)	13% (95% CI: 12–15%)	Limb pain; Type 1: acute, asymmetric, <5 joints; Type 2: symmetric, ≥5 joints	Ultrasound
Ankylosing spondylitis (AS)	3% (95% CI: 2–4%)	Nocturnal lower back pain, improves with exercise, associated with uveitis	MRI (STIR sequences), clinical evaluation

**Table 2:** Summary of selected cutaneous manifestations and complications associated with IBD, including clinical presentation and therapeutic approaches. IBD—inflammatory bowel diseases, TNF-α—tumor necrosis factor alpha, JAK—Janus kinase, BMI—body mass index.

Type of Dermal Manifestation	Dermal Lesion Type	Clinical Presentation	Management
Reactive	erythema nodosum	raised, tender, bright red subcutaneous nodules, most commonly located on the anterior aspect of the lower legs	control of IBD, topical or systemic corticosteroids, TNF-α antagonists
	pyoderma gangrenosum	single or multiple erythematous papules/pustules that progress to necrotic, deep ulcers with irregular borders, most often on the anterior lower legs or periarticular regions	topical and systemic corticosteroids; in advanced cases: cyclosporine, TNF-α antagonists, ustekinumab, or JAK inhibitors
Specific	metastatic Crohn’s disease	abscesses, fistulas, ulcers, nodules occurring on the lower extremities and interdigital areas	topical and systemic corticosteroids, thiopurines, TNF-α antagonists, ustekinumab
Associated	hidradenitis suppurativa	abscesses, nodules in axillary, inguinal, and perianal regions	smoking cessation, BMI reduction, topical clindamycin; systemic: clindamycin + rifampin, retinoids, TNF-α antagonists, ustekinumab, or JAK inhibitors
Complications	TNF-α antagonist–induced psoriasis	erythematous-squamous lesions, psoriatic plaques	discontinuation of TNF-α antagonist, switch to ustekinumab

**Table 3:** Prevalence, clinical features, and diagnostic tools for ocular manifestations in patients with IBD. CD—Crohn’s disease, UC—ulcerative colitis, IBD—inflammatory bowel disease, MTX—methotrexate, TNF-α—tumor necrosis factor alpha, ADA—adalimumab, IFX—infliximab, JAK—Janus Kinase Inhibitors, UST—ustekinumab, NSAID—Non-Steroidal Anti-Inflammatory Drugs.

Type	Prevalence	Clinical Presentation	Diagnostic Approach	Risk of Vision loss	Management
Uveitis	11% in CD; 5.6% in UC	Pain, hyperemia, blurred vision, photophobia; may be bilateral. Usually independent of intestinal disease activity, although an association may occur.	Slit-lamp examination; exclusion of infection; differentiation of uveitis subtype	Present	Urgent ophthalmology consultation. • Anterior uveitis: topical corticosteroids ± mydriatics • Intermediate/posterior/panuveitis: intravitreal or systemic corticosteroids • Persistent/refractory disease: steroid-sparing immunomodulators; ADA or IFX may be considered
Scleritis	<1% of IBD	Severe deep pain, often awakening the patient from sleep; unilateral or bilateral; diffuse or focal scleral hyperemia. Often associated with active IBD.	Does not blanch with 10% phenylephrine; ophthalmologic assessment	Present	Urgent ophthalmology consultation. • NSAIDs or systemic corticosteroids according to severity • Topical/periocular therapy may be considered in selected non-necrotizing cases • Immunomodulators or TNF-α inhibitors for refractory disease
Episcleritis	4% of IBD	Ocular hyperemia with absent or mild discomfort; unilateral or bilateral. Usually parallels intestinal disease activity.	Blanches with 10% phenylephrine; clinical examination	Absent	Treat underlying IBD; artificial tears/topical moisturizing agents and cold compresses. NSAIDs may be considered for more severe symptoms.

**Table 4:** Detailed listing of hepatobiliary manifestations associated with IBD, including characteristic clinical entities, their pathophysiological context, and diagnostic or therapeutic considerations. PSC—Primary Sclerosing Cholangitis, AIH—Autoimmune Hepatitis, PSC-AIH overlap syndrome, MASLD—Metabolic dysfunction-associated steatotic liver disease, HCV—Hepatitis C Virus, HBV—Hepatitis B Virus, EBV—Epstein–Barr Virus, HSV—Herpes Simplex Virus, CMV—Cytomegalovirus, HEV—Hepatitis E Virus, ANA—Antinuclear Antibodies, ASMA—Anti-Smooth Muscle Antibodies, LKM-1—Liver Kidney Microsomal Antibody Type 1, SLA/LP—Soluble Liver Antigen/Liver–Pancreas Antigen, IgG—immunoglobulin G, IgM—immunoglobulin M, MRCP—Magnetic Resonance Cholangiopancreatography, HBsAg—hepatitis B virus surface antigen, anti-HBc—antibody to hepatitis B core antigen, anti-HBs—anti-hepatitis B surface antibody, DNA—deoxyribonucleic acid, RNA—ribonucleic acid, BMI—body mass index, FIB-4—fibrosis-4 index, OGTT—oral glucose tolerance test.

Potential Cause	Management
Autoimmune hepatitis (AIH)	Antibody panel: ANA, ASMA, LKM-1, SLA/LP, total IgG, liver biopsy
Primary sclerosing cholangitis (PSC)	MRCP
Overlap syndrome PSC/AIH, small-duct PSC	MRCP, antibody panel: ANA, ASMA, LKM-1, SLA/LP, total IgG, liver biopsy
Viral hepatitis	Hepatitis A (IgM), Hepatitis B (HBsAg, anti-HBc IgM/total, anti-HBs, HBV DNA), Hepatitis C (anti-HCV, HCV RNA), Hepatitis E (IgM, HEV RNA), CMV (IgM, CMV DNA), EBV (IgM, EBV DNA), HSV (HSV-1 and HSV-2 DNA), Parvovirus (IgM)
Drug-induced liver injury (thiopurines, infliximab, methotrexate)	Discontinue the drug and monitor liver function—if improvement is observed, drug-induced etiology is likely; if not, investigate alternative causes including infectious, autoimmune, or overlap syndromes; consider liver biopsy
Metabolic dysfunction-associated steatotic liver disease (MASLD)	BMI, abdominal ultrasound, assessment of FIB-4 and metabolic parameters (OGTT with insulin curve, full lipid profile, uric acid, homocysteine)

**Table 5:** Relationship between selected extraintestinal manifestations and intestinal IBD activity. IBD—inflammatory bowel disease; SpA—spondyloarthritis.

Extraintestinal Manifestation	Relationship with Intestinal Disease Activity
Peripheral SpA—type 1/oligoarticular	Usually parallels intestinal activity
Peripheral SpA—type 2/polyarticular	Usually independent/variable association
Axial SpA/ankylosing spondylitis	Usually independent
Erythema nodosum	Usually parallels intestinal activity
Pyoderma gangrenosum	Variable; may occur independently
Aphthous stomatitis	Usually parallels intestinal activity
Episcleritis	Usually parallels intestinal activity
Scleritis	Often associated with active intestinal disease
Uveitis	Usually independent, although association with intestinal activity may occur
Primary sclerosing cholangitis	Usually independent

**Table 6:** Practical referral criteria for selected extraintestinal manifestations in patients with inflammatory bowel disease. DETAIL, DETection of Arthritis in Inflammatory boweL diseases; IBIS-Q, IBD Identification of Spondyloarthritis Questionnaire; OMIS, Ocular Manifestations in IBD Screening; eGFR, estimated glomerular filtration rate.

Organ System	Referral Signs/Symptoms	Suggested Action
Musculoskeletal	Inflammatory back pain, persistent peripheral joint pain/swelling, dactylitis, enthesitis	Rheumatology referral; consider DETAIL or IBIS-Q screening
Ocular	Ocular pain, marked redness, photophobia, blurred or reduced vision	Urgent ophthalmology assessment; OMIS may support screening
Dermatological	Painful erythematous nodules, rapidly progressive ulceration, atypical or persistent skin lesions	Dermatology referral
Hepatobiliary	Persistent cholestatic liver test abnormalities, unexplained transaminase elevation, jaundice or pruritus	Hepatology referral
Renal	Persistent haematuria/proteinuria, albuminuria, unexplained decline in eGFR	Nephrology referral
Thromboembolic	Unilateral limb swelling/pain, sudden dyspnoea, pleuritic chest pain, haemoptysis	Immediate diagnostic assessment

**Table 7:** Therapeutic evidence for selected classical extraintestinal manifestations in inflammatory bowel disease. SpA—spondyloarthritis; EN—erythema nodosum; PG—pyoderma gangrenosum; EIM—extraintestinal manifestation.

Treatment	Peripheral SpA	Axial SpA	EN/PG	Ocular EIMs
Infliximab	Evidence of benefit	Evidence of benefit	Strongest evidence for PG; benefit reported for EN	Evidence of benefit, particularly in refractory uveitis
Adalimumab	Evidence of benefit	Evidence of benefit	Limited/observational evidence	Evidence of benefit in uveitis
Golimumab	Limited evidence in IBD-associated disease	Indirect evidence from axSpA	Insufficient EIM-specific evidence	Insufficient EIM-specific evidence
Vedolizumab	Mixed evidence; new or worsening joint symptoms reported	No established efficacy; possible worsening in some patients	Limited evidence	Limited evidence
Ustekinumab	Possible benefit; evidence limited	Efficacy not established	Case reports/limited evidence, particularly for PG	Limited evidence
Tofacitinib	Limited IBD-specific evidence	Indirect evidence from axial SpA/AS studies	Case reports/limited evidence	Case reports/insufficient evidence
Upadacitinib	Limited IBD-specific evidence	Indirect evidence from axSpA/AS trials	Emerging evidence	Limited evidence
Filgotinib	Insufficient EIM-specific evidence	Indirect evidence from SpA studies	Insufficient evidence	Insufficient evidence
Ozanimod	No established EIM-specific efficacy	No established efficacy	Insufficient evidence	Insufficient evidence
Mirikizumab	Emerging post hoc evidence	Emerging/limited evidence	Emerging/limited evidence	Emerging post hoc evidence
Risankizumab	Insufficient EIM-specific evidence	No established EIM-specific efficacy	Insufficient evidence	Insufficient evidence
Guselkumab	Insufficient IBD-EIM-specific evidence	Insufficient IBD-EIM-specific evidence	Insufficient IBD-EIM-specific evidence	Insufficient evidence

## Data Availability

No new data were created or analyzed in this study. Data sharing is not applicable to this article.

## Abbreviations

ACD	anemia of chronic disease
ACPA	anti-citrullinated protein antibodies
ACR	albumin-to-creatinine ratio
ADA	adalimumab
AD	atopic dermatitis
AIH	autoimmune hepatitis
ANA	antinuclear antibodies
anti-HBc	antibody to hepatitis B core antigen
anti-HBs	antibody to hepatitis B surface antigen
anti-HCV	antibody to hepatitis C virus
AS	ankylosing spondylitis
ASMA	anti-smooth muscle antibodies
ATs	advanced therapies
ax SpA	axial spondyloarthritis
AZA	azathioprine
BMI	body mass index
CD	Crohn’s disease
CKD	chronic kidney disease
CMV	cytomegalovirus
CRP	C-reactive protein
CU	ulcerative colitis (colitis ulcerosa)
DNA	deoxyribonucleic acid
EBV	Epstein–Barr virus
ECCO	European Crohn’s and Colitis Organisation
EIM	extraintestinal manifestation
EN	erythema nodosum
ERCP	endoscopic retrograde cholangiopancreatography
ETRA	etrasimod
ETZ	etrolizumab
FIB-4	fibrosis-4 index
FILGO	filgotinib
GFR	glomerular filtration rate
GOL	golimumab
GUS	guselkumab
HBsAg	hepatitis B surface antigen
HBV	hepatitis B virus
HCV	hepatitis C virus
HEV	hepatitis E virus
HLA	human leukocyte antigen
HS	hidradenitis suppurativa
IBD	inflammatory bowel disease
IFX	infliximab
IgA	immunoglobulin A
IgG	immunoglobulin G
IgM	immunoglobulin M
IL	interleukin
IL-12/23	interleukin 12 and interleukin 23
IL23R	interleukin 23 receptor
JAK	Janus kinase
LKM-1	liver kidney microsomal antibody type 1
LPS	lipopolysaccharide
MASLD	metabolic dysfunction-associated steatotic liver disease
MAdCAM-1	mucosal addressin cell adhesion molecule-1
MCD	metastatic Crohn’s disease
MIRI	mirikizumab
MRCP	magnetic resonance cholangiopancreatography
MRI	magnetic resonance imaging
MTX	methotrexate
NOD2/CARD15	nucleotide-binding oligomerization domain-containing protein 2/caspase recruitment domain-containing protein 15
Non-axSpA	non-axial spondyloarthropathy
Nr-axSpA	non-radiographic axial spondyloarthritis
NSAID	non-steroidal anti-inflammatory drug
OGTT	oral glucose tolerance test
OTM	ontamalimab
pSpA	peripheral spondyloarthritis
PG	pyoderma gangrenosum
PML	progressive multifocal leukoencephalopathy
PSC	primary sclerosing cholangitis
PsA	psoriatic arthritis
QoL	quality of life
RA	rheumatoid arthritis
RISA	risankizumab
RF	rheumatoid factor
RNA	ribonucleic acid
SCFA	short-chain fatty acid
SIBDCS	Swiss IBD Cohort Study
SLA/LP	soluble liver antigen/liver-pancreas antigen
SpA	spondyloarthropathy
SPIRIT	Selecting End Points for Disease-ModIfication Trials
STIR	short tau inversion recovery (MRI sequence)
STRIDE II	Selecting Therapeutic Targets in Inflammatory Bowel Disease II
Tc	tacrolimus
TIMP3	tissue inhibitor of metalloproteinases 3
TNF-α	tumor necrosis factor alpha
TOF	tofacitinib
UC	ulcerative colitis
UDCA	ursodeoxycholic acid
UNITI	ustekinumab clinical trial program
UPA	upadacitinib
US	ultrasound
UST	ustekinumab
VDZ	vedolizumab
VEGF	vascular endothelial growth factor
VTE	venous thromboembolism
